# Fiske Prize Fund—Rhode Island

**Published:** 1856-09

**Authors:** 


*Fiske Prize Fund—Rhode Island.*

We cheerfully comply with the request of Dr. Parsons to insert the following notice. We trust that some of our readers in this and other States of the Union will enter the lists to which they are so cordially invited with the profession of Rhode Island.—*Med. & Surg. Reporter.*

The Trustees of the Fiske Fund, at the late meeting of the Rhode Island Medical Society, offered a Premium of One Hundred Dollars for the best Dissertation on the following question:—"What are the causes and nature of that disease, incident to pregnancy and lactation, characterized by inflammation and ulceration of the mouth and fauces, usually accompanied by anorexia, emaciation, and diarrhœa—and what is the best mode of treatment?"

They announce that such dissertations should be sent, free of expense, to their Secretary, S. A. Arnold, M.D., Providence, R.I., by the first of May, 1857; each dissertation having a motto inscribed on it, and the same motto on a sealed packet, containing the name and address of the writer inside. A condition of receiving the premium is that the property in the dissertation must first be transferred to the trustees of the fund, who cause all successful dissertations to be printed and distributed.

The prize for 1856, on the subject, "Does pregnancy accelerate or retard the development of tubercle of the lungs in persons predisposed to that disease?" was awarded to Dr. Edward Warren, of Edenton, North Carolina.

